# Pandemic (H1N1) 2009–associated Pneumonia in Children, Japan

**DOI:** 10.3201/eid1702.091904

**Published:** 2011-02

**Authors:** Maki Hasegawa, Takafumi Okada, Hiroshi Sakata, Eiichi Nakayama, Tatsuo Fuchigami, Yasuji Inamo, Hideo Mugishima, Takeshi Tajima, Satoshi Iwata, Miyuki Morozumi, Kimiko Ubukata, Haruo Watanabe, Takashi Takahashi

**Affiliations:** Author affiliations: Kitasato University, Tokyo (M. Hasegawa, T. Okada, E. Nakayama, M. Morozumi, K. Ubukata, T. Takahashi);; Nihon University School of Medicine, Tokyo, Japan (M. Hasegawa, T. Fuchigami, Y. Inamo, H. Mugishima);; National Hospital Organization Tokyo Medical Center, Tokyo (T. Okada, S. Iwata);; Asahikawa-Kosei General Hospital, Asahikawa, Japan (H. Sakata);; Komagome Hospital, Tokyo (E. Nakayama);; Hakujikai Memorial Hospital, Tokyo (T. Tajima);; National Institute of Infectious Diseases, Tokyo (H. Watanabe)

**Keywords:** Pandemic (H1N1) 2009 virus, viruses, influenza, pediatric inpatients, pneumonia, children, complicating illnesses, Japan, dispatch

## Abstract

To describe clinical aspects of pandemic (H1N1) 2009 virus–associated pneumonia in children, we studied 80 such children, including 17 (21%) with complications, who were admitted to 5 hospitals in Japan during August–November 2009 after a mean of 2.9 symptomatic days. All enrolled patients recovered (median hospitalization 6 days). Timely access to hospitals may have contributed to favorable outcomes.

We describe the clinical aspects of pandemic (H1N1) 2009 virus infection in children who developed spontaneous pneumomediastinum (*1*) or plastic bronchitis (*2*). In Mexico, 18 persons, including 5 children, had pandemic (H1N1) 2009–associated pneumonia (*3*). However, active surveillance to collect data on pneumonia cases among children infected with pandemic (H1N1) 2009 virus has not been conducted in Japan.

## The Study

Active procurement of specimens from pediatric inpatients with pandemic (H1N1) 2009–associated pneumonia was organized by the Laboratory of Molecular Epidemiology for Infectious Agents at Kitasato University. Clinical data and respiratory specimens were provided by pediatric departments at 5 institutions during August 9–November 6, 2009. Pandemic (H1N1) 2009–associated pneumonia was diagnosed from influenza-like illnesses associated with infiltrates on chest radiographs and laboratory-confirmed pandemic (H1N1) 2009 virus (*3*). Each patient’s pediatrician informed us of any major complication that followed the pneumonia.

First, patients were divided into 2 groups: those who had and did not have complications. The group having no complications then was divided into 2 age-defined subgroups (cutoff, 6 years). Each subgroup was further divided into subgroups: hospital admission 1–3 days after symptom onset or admission >4 days after symptom onset. Information about clinical features; routine laboratory findings at hospital admission; and if available, serum immunoglobulin E concentration was obtained from patients’ medical charts. Tachypnea was defined by using criteria in Japanese guidelines adopted in 2007 for managing respiratory infectious diseases (*4*) in children. Chest radiographic findings taken at time of hospital admission were classified by extent of pulmonary infiltrates (localized vs. diffuse) and infiltrate distribution (bilateral vs. unilateral; upper, middle, or lower lung field) (*4*).

Nasopharyngeal swabs (n = 79) or an endotracheal aspirate were sent to the laboratory for microbiologic identification. Pandemic (H1N1) 2009 virus in specimens was determined by real-time reverse transcription–PCR (RT-PCR) (*1**,**2*). Additionally, comprehensive real-time RT-PCR was performed to confirm respiratory co-infection with any of 12 viruses (*5*). Multiplex real-time PCR also was performed to detect 6 respiratory bacteria (*6*).

Patient demographic characteristics, symptoms, physical findings, treatments, and clinical courses were compared between groups with and without complications by using the χ^2^ test. Neutrophil and lymphocyte counts were analyzed by using box-and-whisker plots. A p value <0.05 indicated a significant difference between patient groups.

The study comprised 80 pediatric inpatients who received treatment at 5 medical institutions for pandemic (H1N1) 2009–associated pneumonia over a 3-month period. Family members were informed about the purpose of the study, and children’s parents provided informed consent.

We compared patients by presence or absence of complications (Table 1). Complications included pleural effusion (5 patients), pneumomediastinum (6), atelectasis (6), myositis (2), and plastic bronchitis (1). No patients had organ dysfunction or encephalopathy.

**Table 1:** Demographic characteristics and clinical features of children hospitalized with pandemic (H1N1) 2009–associated pneumonia, Japan, August–November 2009*

Variable	Total	Group A, no complications, n = 63	Group B, complications, n = 17	p value, A vs. B
Sex, M/F	57 (71.3)/23 (28.8)	46 (73.0)/17 (27.0)	11 (64.7)/6 (35.3)	0.71
Median age, y (range)	7 y (9 mo–14y)	7 y (9 mo–14 y)	6 y (4 y–12 y)	0.41
<1 y	4 (5)	4 (6.3)	0	
2–5 y	14 (17.5)	11 (17.5)	3 (17.6)	
>6 y	62 (77.5)	48 (76.2)	14 (82.3)	
History of asthma	26 (32.5)	21 (33.3)	5 (29.4)	0.76
Admission <3 d/>4 d after symptom onset	61 (76.3)/19 (23.8)	45 (71.4)/18 (28.6)	16 (94.1)/1 ( 5.9)	0.10
Major symptoms, physical findings				
Cough	66 (82.5)	53 (84.1)	13 (76.5)	0.71
Respiratory distress	29 (36.3)	17 (27.0)	12 (70.6)	<0.01
Fever >38°C	74 (92.5)	58 (92.1)	16 (94.1)	0.53
Tachypnea	57 (71.3)	42 (66.6)	15 (88.2)	0.15
Inspiratory retraction	39 (48.8)	27 (42.9)	12 (70.6)	0.04
Rhonchi	48 (60.0)	40 (63.5)	8 (47.1)	0.22
SpO_2_ <93	39 (48.8)	25 (39.7)	14 (82.4)	<0.01
Treatment and clinical course				
O_2_ supplementation	49 (61.3)	34 (54.0)	15 (88.2)	0.01
Mean duration of O_2_ administration, d (range)	3.5 (1–11)	2.9 (1–6)	4.7 (1–11)	0.02
Treatment with oseltamivir	67 (83.8)	51 (80.9)	16 (94.1)	0.35
Treatment with antimicrobial drugs†	63 (78.8)	46 (73.0)	17 (100)	0.04
Isoproterenol inhalation	6 (7.5)	0	6 (35.3)	<0.01
Median duration of hospitalization, d (range)	6 (3–18)	6 (3–9)	8 (5–18)	<0.01

*Values are no. (%) except as indicated. SpO_2_, percutaneous oxygen saturation while breathing room air. †Parenteral infusion of sulbuctum/ampicillin or cefazoin was carried out for 3–5 d.

The median age of pneumonia patients was 7 years; 57 (71%) were male; 26 (33%) had asthma, 4 (5%) had atopic dermatitis without asthma, and 1 (1%) had DiGeorge syndrome. Forty-nine (61%) patients were previously healthy. Mean time from onset of illness to admission was 2.9 days; 61 (76%) patients were admitted early to the hospital (within 3 days after symptom onset). Respiratory distress, inspiratory retraction, and low percutaneous oxygen saturation (<93% while breathing room air) were significantly more frequent among patients with than without complications (p<0.01).

Infiltrates were more often localized (64 patients) than diffuse (16 patients). Unilateral localized infiltrates occurred more commonly in a lower lung field than in upper or middle fields, and unilateral infiltrates were more common in the right than left lung.

**Figure 1:**
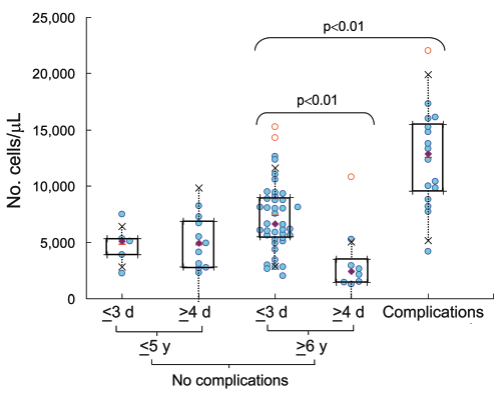
Neutrophil counts (cells/μL) in blood samples from 5 groups: patients with complications, patients >6 years of age without complications who had early or late hospital admission, and patients <5 years of age without complications who had early or late hospital admission. Data were analyzed by using box-and-whisker plots. Lower limit, median, and upper limit shown within each box correspond to the 25%, 50%, and 75% percentile, respectively; half of the patients considered fall within each box. Dotted lines extending from each box represent 1.5× the quartile deviation. Open red circles, outlying cases; closed diamonds, medians; horizontal bars, means.

**Figure 2:**
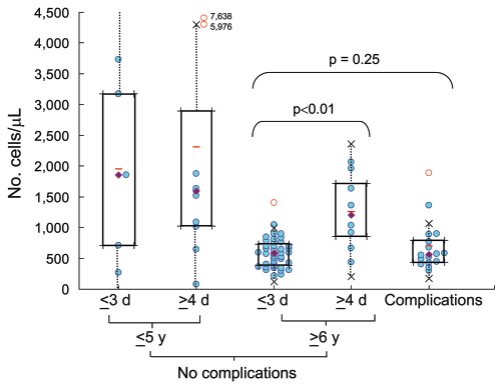
Lymphocyte counts (cells/μL) in blood samples from 5 groups (patients with complications, patients >6 years of age without complications who had early or late hospital admission, and patients <5 years of age without complications who had early or late hospitalization). Data were analyzed by using box-and-whisker plots. Lower limit, median, and upper limit shown within each box correspond to the 25%, 50%, and 75% percentile, respectively; half of the patients considered fall within each box. Dotted lines extending from each box represent 1.5× the quartile deviation. Open red circles, outlying cases; closed diamonds, medians; horizontal bars, means.

Clinical laboratory results are shown in Table 2. The neutrophil count was significantly higher in patients with complications than in others (Figure 1). Lymphopenia (<1,000 cells/μL) was characteristic in children with complications and in children who had no complications and were >6 years of age and admitted to the hospital on day 1–3 of illness (Figure 2). Lymphocyte count was significantly higher in the corresponding group with admission >4 days after onset. Serum immunoglobulin E concentration was high (>170 IU/mL) in both groups admitted on day 1–3, regardless of whether complications were present.

**Table 2:** Laboratory test results for children hospitalized with pandemic (H1N1) 2009–associated pneumonia, Japan, August–November 2009*

Characteristic	Group A, no complications, n = 63			Group B, complications, n = 17
	Age <5 y, n = 15	Age >6 y, n = 48		Age >6 y, n = 14
Leukocytes, cells/μL (range)†	6,400 (3,600–14,400)	7,400 (2,400–17,100)		14,200 (5,100–22,700)
Neutrophils‡	4,929 (2,227–8,256)	6,081(1,248–15,287)		12,849 (4,182–22,042)
Lymphocytes§	1,593 (74–7,638)	608 (214–2,064)		560 (295–1,889)
Eosinophils	0 (0–102)	16 (0–918)		23 (0–145)
Monocytes	240 (37–1,685)	359 (99–1,271)		337 (0–714)
CRP, mg/dL (range)¶	1.0 (0.1–2.9)	2.4 (0.05–11.95)		3.5 (1–7.83)
LDH, IU/L (range)¶	304 (230–415)	248 (182–575)		248 (193–353)
CK, IU/L (range)¶	101 (40–328)	110 (29–2,240)		148 (57–1,524)
IgE, IU/mL (range)#	61 (7.3–311)	443 (34–4,680)		1,058 (43–4,011)

*CRP, C-reactive protein; LDH, lactate dehydrogenase; CK, creatine kinase; Ig, immunoglobulin. †Reference ranges by age group: <1 y, 7,000–15,000; 2–5 y, 7,000–11,000; >6 y, 6,500–10,000. ‡Reference range by age group: <1 y, 4,000–8,000; 2–5 y, 2,500–5,500; >6 y, 3,000–5,000. §Reference range by age group: <1 y, 4,000–11,000; 2–5 y, 3,000–7,000; >6 y, 2,500–4,500. ¶Reference upper limits for CRP, LDH, and CK levels are 0.3, 400, and 200, respectively. #Reference ranges for IgE by age group: 1–3 y, <30; 4–6 y, <110; >7 y, <170. Serum IgE data were analyzed when available.

PCR detected bacteria in nasopharyngeal specimens from 41 (51%) patients. Organisms present included *Streptococcus pneumoniae* (25 patients), *Haemophilus influenzae* (28), and *Mycoplasma pneumoniae* and *S. pyogenes* (1 each); some patients had multiple organisms. In addition, rhinovirus was detected in 2 patients and enterovirus in 1.

Forty-nine (61%) patients required oxygen administration (mean duration 3.5 days) (Table 1). Oxygen supplementation was provided significantly more often to children who had than who did not have complications (15 [88%] vs. 34 [54%]; p<0.05). A total of 67 (84%) patients received oseltamivir, and 63 (79%) received antimicrobial drugs. Median time from onset of symptoms to initiation of oseltamivir treatment (4 mg/kg/d for 5 days) based on 20 applicable patients was 2 days, showing no differences between groups. Isoproterenol inhalation was needed only for patients with complications. In 1 patient who had an asthma attack, plastic bronchitis developed and the patient required invasive mechanical ventilation for 5 days.

All children recovered, with a median hospital stay of 6 days (Table 1). Hospitalization was longer for patients with than without complications (median 8 days vs. 6 days; p<0.01).

Our study has several limitations. Our PCR data from nasopharyngeal swabs cannot distinguish pathogens from colonizing organisms and cannot reliably guide decisions regarding antimicrobial drug treatment. Various reports have described invasive secondary bacterial infection with *Staphylococcus* *aureus* diagnosed from lower respiratory tract or blood specimens (*7**,**8*); such cultures were not obtained from all of the patients in our study. Moreover, pneumonia may have been underdiagnosed in our patients considering limited sensitivity of chest radiography compared with computed tomography (*9*).

## Conclusions

Pediatricians should be aware that early diagnosis of influenza can enable prompt antiviral treatment of severe illness. All Japanese citizens have ready access to medical institutions through the national health insurance system. On November 13, 2009, the Japan Pediatric Society reported surveillance data concerning 60 pandemic (H1N1) 2009–associated deaths in children (*10*). Main causes of death were sudden death and rapidly progressive severe pneumonia. Testing practices, access, and policies regarding early administration of antiviral agents have protected many children from life-threatening pandemic (H1N1) 2009.
